# The risk factors of postoperative cognitive dysfunction in patients undergoing carotid endarterectomy: an updated meta-analysis

**DOI:** 10.1186/s13019-023-02428-6

**Published:** 2023-11-09

**Authors:** Jinhua He, Ran Duan, Peng Qiu, Huanhuan Zhang, Meng Zhang, Meinv Liu, Xiaoqian Wu, Jianli Li

**Affiliations:** 1 Department of Anesthesiology, Hebei General Hospital, Shijiazhuang, China; 2 Clinical Laboratory, Hebei General Hospital, Shijiazhuang, 050051 China

**Keywords:** Postoperative cognitive dysfunction, Carotid endarterectomy, Risk factor, Meta-analysis

## Abstract

**Objective:**

The purpose of the current meta-analysis was to determine the incidence and risk factors to provide a scientific basis for prevention and treatment of postoperative cognitive dysfunction (POCD) after carotid endarterectomy (CEA).

**Methods:**

Relevant articles published before October 2022 were searched from Pubmed/MEDLINE, Cochrane and Embase databases. The outcomes were the incidence and risk factors for POCD. A random-effects model was applied to estimate the overall odds ratios (ORs) and mean differences (MDs) for all risk factors through STATA 14.0 and RevMan 5.4. The quality of eligible studies was evaluated by Newcastle–Ottawa Scale (NOS) as previously described.

**Results:**

A total of 22 articles involving 3459 CEA patients were finally identified. The weighted mean incidence of POCD was 19% (95% confidence intervals (95% CI) 0.16–0.24, *P* < 0.001). Of the 16 identified risk factors, hyperperfusion (OR: 0.54, 95% CI 0.41–0.71) and degree of internal carotid artery (ICA) stenosis (OR: 5.06, 95% CI 0.86–9.27) were the potential risk factors of POCD, whereas patients taking statins preoperative had a lower risk of POCD (OR: 0.54, 95% CI 0.41–0.71). Subgroup analysis revealed that the risk of POCD at 1 month after CEA was higher in patients with diabetes (OR: 1.70, 95% CI 1.07–2.71).

**Conclusion:**

The risk factors of POCD were hyperperfusion and degree of ICA stenosis, while diabetes could significantly increase the incidence of POCD at 1 month after surgery. Additionally, preoperative statin use could be a protective factor for POCD following CEA.

**Supplementary Information:**

The online version contains supplementary material available at 10.1186/s13019-023-02428-6.

## Introduction

Stroke is the most common cause of disability and death in the world, killing about 12 million people each year [1]. The carotid artery is one of the most common sites of atherosclerosis, and 30% of ischemic strokes originate in the carotid artery [2]. CEA is one of the main effective interventions for patients with severe carotid stenosis to reduce stroke, which is associated with various postoperative complications, including POCD. POCD, defined as a significant decrease in cognitive ability after surgery or anesthesia, is mainly associated with serious surgical outcomes, overall declined quality of life, prolonged hospitalization and even increased mortality [3]. Recently, a study indicated that the incidence of POCD in patients undergoing CEA was from 6 to 30%, and cognitive decline at 3 months in patients might be a risk factor for poor long-term survival [4]. Therefore, systematic identification of risk factors is essential for doctors and nurses to develop prevention strategies to reduce the incidence of POCD following CEA.

Different from other surgery, the mechanism of POCD after CEA might be closely associated to existed cerebrovascular lesions and the specificity of the surgical procedure. Recently, Arba et al. [5] found that cerebral small vessel disease might play a relevant role in developing cognitive impairment after CEA. A previous study indicated that the occurrence of POCD was related to covert stroke caused by cerebral microembolism during surgery [6]. Moreover, several studies confirmed that extension of cross-clamping duration and postoperative hyperperfusion could be associated with the incidence of POCD following CEA [7, 8]. There is accumulating evidence indicating that POCD is complex and multifactorial, the interaction between predisposing and precipitating factors plays a vital role in developing POCD. A meta-analysis by Aceto et al. [9] first reported the risk factors of POCD in patients undergoing CEA, while the limitations stemmed from the heterogeneity of the included studies, such as cognitive function assessment and cohort size. Furthermore, several new observational studies reported risk factors of cognitive decline after CEA recently [4, 6, 10]. Therefore, it is necessary to update the meta-analysis of POCD-related risk factors following CEA.

In the present meta-analysis, we will systematically review the incidence and perioperative risk factors for POCD following CEA by comparing with the prior articles, to provide more accurate guidance for routine screening risk factors, which may be beneficial to early interventions of at-risk individuals.

## Materials and methods

### Search strategy

The present meta-analysis was based on the Preferred Reporting Items for Systematic Reviews and Meta-Analyses (PRISMA) statement [11], which was performed following a pre-established protocol registered on PROSPERO (CRD 42023388096). A systematic search was conducted on PubMed/MEDLINE, the Cochrane Library and Embase database for all relevant articles published from the database inception to October 25, 2022. The search strategy was based on the search components as follows: “carotid endarterectomy” and “cognitive function”, which also composed of all relevant words to these search terms through the MeSH database and expert opinions (refer to search strategy details in Additional file 1: Appendix 1).

### Study selection

All original English prospective and retrospective studies including cohort, case–control, cross-sectional, etc. were considered qualified for inclusion criteria, which assessed cognitive functions in patients before and after CEA. We excluded review articles, meta-analyses, conference abstracts, comments and case reports/case series. Moreover, the reported outcomes should be odd ratios (ORs) of risk factors with 95% confidence intervals (95% CI).

All records containing titles and abstracts were inputted into Endnote X9 and repeating items were expurgated. Then, we screened titles and abstracts of the remaining studies according to inclusion criteria. The full texts of potentially included articles were further obtained and assessed by two reviewers to identify studies meeting the selection criteria independently. Any different opinions were identified by discussion, with the participation of a third author if necessary. In addition, all relevant reviews screened by the original search and the reference lists were assessed for additional eligible studies.

### Data extraction and quality evaluation

We extracted details of the eligible studies, such as the first name of author, study type, year and country of publication, patient characteristics (mean or median age, percentage of female, percentage of preoperatively symptomatic patients and diabetes), methodological standards (cognitive assessment criteria and time of preoperative and postoperative cognitive assessment), Outcome measures(sample size, ORs or MD with 95% confidence intervals of risk factors and incidence of POCD when reported). Variables represented by median and quartile were converted to mean standard deviation [12]. All data was extracted by two reviewers independently, and disagreements were resolved by discussion (with a third author if necessary). The quality of eligible articles was assessed through the Newcastle–Ottawa scale (for cohort studies) [13].

### Data synthesis and statistical analysis

POCD incidence was shown as proportion of the case in total sample and pooled as proportional-weighted estimates. Review Manager 5.4 and STATA 14.0 software were used to conduct the meta-analysis. Odds ratios (ORs) with 95% confidence intervals (CIs) were applied to express effect-size for dichotomous data, while mean difference (MD) and 95%CIs were used to show continuous data. If data on risk factors for studies was incomplete, the effect estimate was shown as log[OR] with standard error. First, we performed a heterogeneity test on identified studies through *I*^2^ test. A fixed effects model was applied to conduct the meta-analysis if there was no heterogeneity (*P* > 0.1 or *I*^2^ < 50%) in the eligible studies. If there was significant heterogeneity (*P* < 0.1 or *I*^2^ ≥ 50%), a random effects model was applied for the meta-analysis. Publication bias was assessed as quality through Egger’s tests. A value of *P* < 0.05 was identified statistically significant.

## Results

### Study selection

**Fig. 1:**
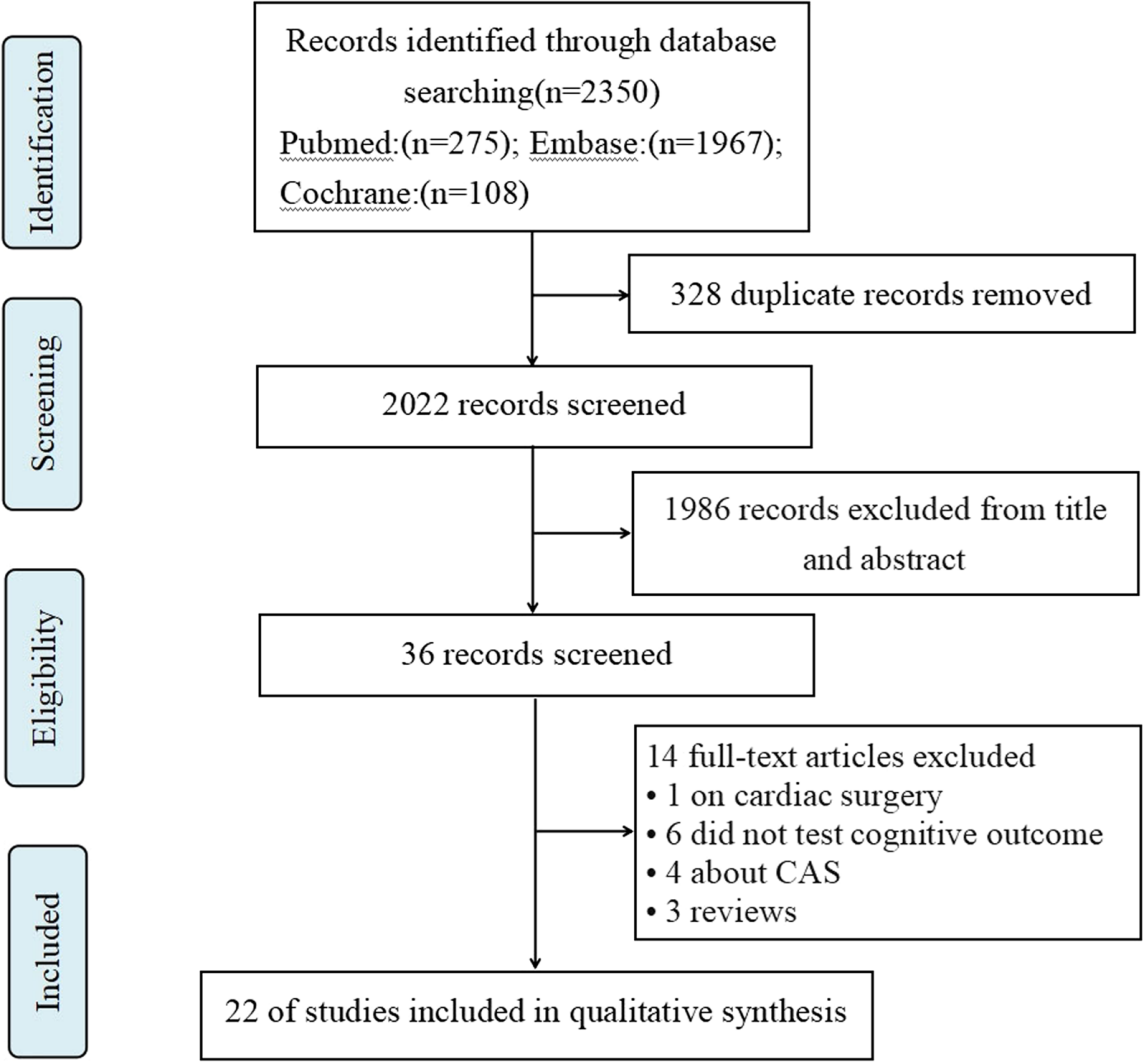
Flow diagram of study select

The original literature searched from three databases produced 2350 records. 2022 studies remained when 328 duplicate records were removed. Then, 1986 studies excluded according to titles and abstracts, and the full texts of 36 relevant studies were accessed and reviewed. Finally, a total of 22 observational studies, published between 2001 and 2021, were identified for further qualitative and quantitative synthesis. The database screening process was shown in Fig. 1.

### Study characteristics and quality evaluation

The characteristics of the eligible articles were shown in Table 1. 22 prospective and retrospective studies, including a total of 3459 adult patients undergoing CEA. Among them, 20 articles were cohort studies and 2 were case–control studies. A total of 9 studies (42.9%) assessed the cognitive function at 3 days after surgery, 8 studies (38.1%) at one month and 4 studies (19%) at one year after surgery. The sample size of these eligible articles ranged from 36 to 585. The mean age of participants was 68.39 ± 8.03 years old, and the ratio of male to female participants was 2528: 931. For quality evaluation, all eligible studies with NOS scores were more than 6, so the quality of these eligible articles was reliable (studies with NOS scores details can be found in Additional file 1: Appendix 3).

**Table 1:** Characteristics of observational studies included in the meta-analysis (n = 22)

Study	Country	CEA, n	Symptomatic patients, n (%)	DM, n (%)	Age, mean (SD)	Female gender, n (%)	Investigated Domains	POCD Assessment criteria	Time of Preoperative Assessment	Time of POCD Assessment
Zhang et al. [10]	China	84	24 (57.14)	24 (28.57)	65.05 (6.67)	20 (23.81)	CIS	z scores	Before surgery	1–2 ds
Igarashi et al. [8]	Poland	75	47 (62.67)	26 (34.67)	69 (6)	2 (2.67)	VSA, LR-IQ, M	SD	3 ds	2 ms
Relander et al. [4]	Finland	43	21 (48.8)	23 (53.49)	–	15 (34.88)	EF, M, LR-IQ	z scores	1–2 ds	3 ms
Robison et al. [34]	USA	576	232 (40.2)	123 (21.4)	70.7 (8.6)	200 (34.8)	EF, M, LR-IQ, VSA	z scores	24 h	24 hs
Oksana et al. [7]	Russian	446	361 (77)	109 (23)	63 (57–69)	147 (31.54)	CIS	SD	1 day	24 hs
Zhang et al. [20]	China	36	-	25 (69)	67 (9)	11 (30.5)	CIS, EF, VSA	SD	Within 3 ds	3 ds and 3 ms
Heyer et al., 2015 [35]	USA	585	237 (40.5)	125 (21.4)		206 (35.2)	EF, M, A, VSA	z scores	Before surgery	24 hs and 1 m
Heyer et al. [36]	USA	411	171 (41.6)	85 (20.7)	–	139 (33.8)	EF, M, A, VSA	z scores	Before surgery	24 hs and 1 m
Sussman et al. [37]	USA	38	18 (60.5)	2 (5.2)	–	16 (42.1)	EF, M, L, VSA	z scores	Before surgery	1d
Heyer et al. [38]	USA	162	−	–	–	–	M, VSA, EF	z scores	Before surgery	1d
Saito et al. [39]	Japan	100	64 (64)	37 (37)	68 (6)	11 (11)	VSA, LR-IQ, M	SD	Within 7 ds	1 m
Takahashi et al. [40]	Japan	37	37 (100)	9 (24)	78.4 (2.2)	4 (10)	VSA, LR-IQ, M	SD	7ds	7ds
Yoshida et al., 2012 [41]	Japan	213	138 (64.8)	71 (33.3)	68.1 (5.8)	15 (7)	LR-IQ, M, VSA	SD	Within 7 ds	1–2 ms
Nanba et al. [17]	Japan	70	50 (71.4)	27 (38.7)	67.9 (7.6)	7 (10)	LR-IQ, M	SD	Before surgery	1 m
Gaudet et al. [42]	USA	64	9 (14.1)	11 (17.1)	73	17 (26.6)	L, M, A, EF	z score	Before surgery	24 hs
Chida et al. [43]	Japan	60	37 (61.7)	21 (35)	68.6 (6.8)	4 (6.7)	VSA, LR-IQ, M	SD	Before surgery	1 m
Soinne et al., 2009 [44]	Finland	44	21 (47.7)	8 (35)	65.3 (8.4)	16 (36.4)	L, M, A, EF	z scores	1 d	5 ds, 102 ds
Hirooka et al. [18]	Japan	158	111 (70.2)	–	67.2 (6.5)	8 (5)	VSA, LR-IQ, M	SD	Before surgery	1 m
Mocco et al. [28]	USA	153	63 (41.2)	38 (24.8)	69.8 ± 8.5	47 (30.7)	L, EF, A, VSA	z scores	Before surgery	1 d and 1 m
Ogasawara et al. [45]	Japan	92	50 (54.3)	31 (33.7)	67.7 (5.5)	11 (12)	VSA, LR-IQ, M	SD	1 m	1 m
Sahlein et al. [46]	USA	43	21 (48.8)	11 (26)	70.2 (8.2)	15 (35)	A, EF, L, VSA	z scores	–	1d
Connolly et al. 47]	USA	53	18 (34)	12 (22.6)	–	20 (37.7)	L, EF, A, VSA	z scores	Before surgery	1d

*DM* diabetes mellitus; *d/ds* day/s; *m/ms* month/s; *ws* weeks; *hs* hours; *SD* standard deviation; *CIS* cognitive impairment screening; *M* memory; *L* language; *EF* executive functioning; *A* attention; *LR-IQ* logical reasoning and global intelligence; *VSA* visuo-spatial abilities

### POCD incidence rate

**Fig. 2:**
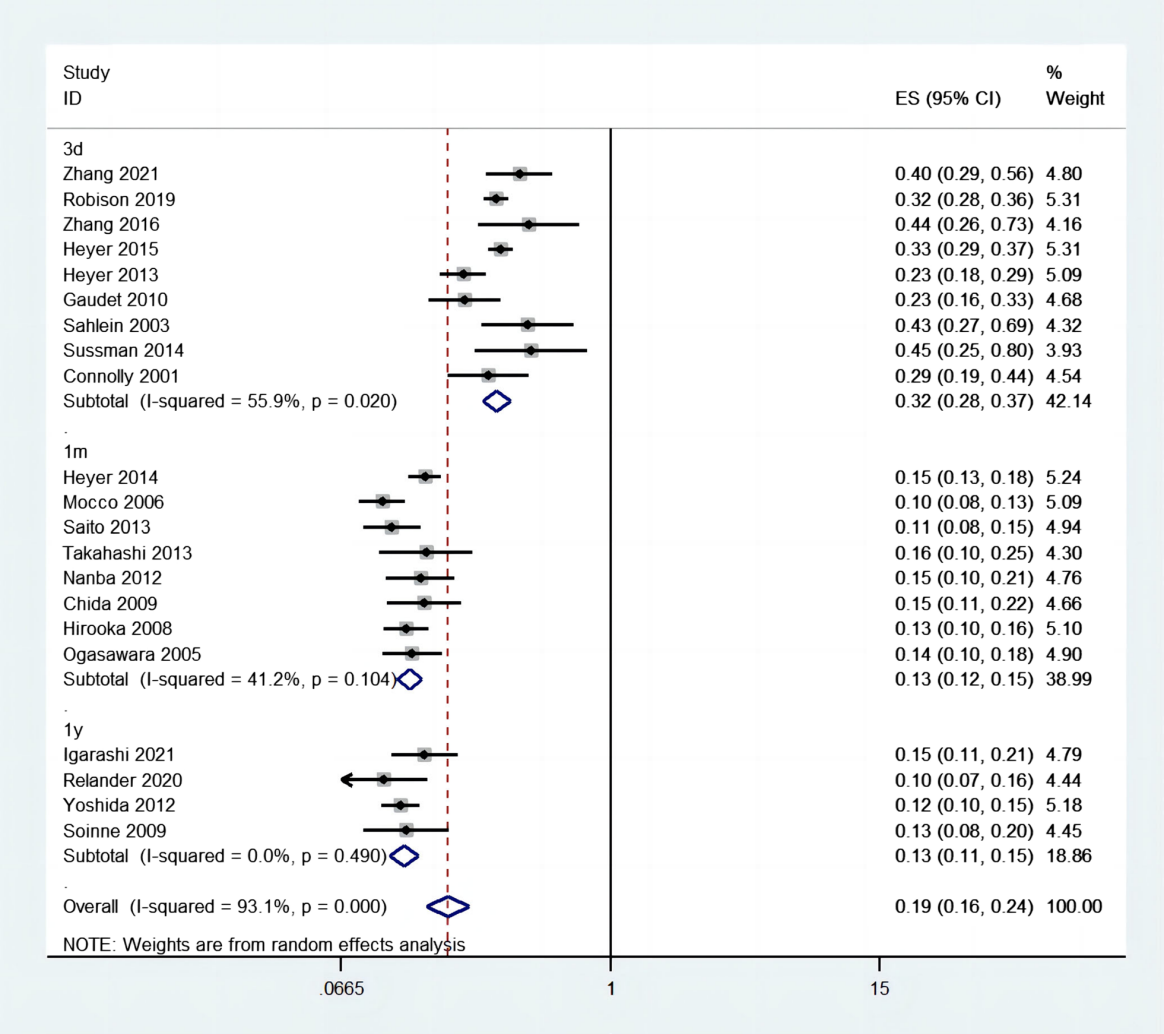
Forest plot of studies (n = 21) evaluating POCD incidence after CEA

Incidence of POCD was reported in 21 studies, in total of 3413 patients. The weighted mean incidence of POCD was 19% (95% CI 0.16–0.24), as indicated in Fig. 2. Nevertheless, the result of synthesized incidence lacked reliability due to heterogeneity was significant (*I*^2^ = 93.1%, *P* < 0.001). The result of subgroup analysis showed that incidence of POCD at 3 days, 1 month, 1 year after surgery was 32% (*I*^2^ = 55.9%, 95% CI 0.28–0.37, *P* = 0.02), 13% (*I*^2^ = 41.2%, 95% CI 0.12–0.15, *P* = 0.104), 13% (*I*^2^ = 0%, 95% CI 0.11–0.15, *P* = 0.49), respectively.

### Patient-related risk factors for POCD

The pooled odds ratios (ORs) and mean difference (MD) of 16 risk factors with their heterogeneity test and confidence intervals results were summarized in Table 2.

**Table 2:** Results of meta-analysis on patient-related and procedure-related risk factors for POCD (continuous and dichotomous variables)

Risk factors	No. of patients (POCD/no POCD)	No. of included studies	MD	Lower 95% CIs	Upper 95% CIs	*I*^2^ (%)	*P* value
Age (years)	163/917	13	0.65	− 0.40	1.70	0	0.22
Cross-clamping duration (min)	100/699	9	0.86	− 0.55	2.27	11	0.23
Education years	58/176	5	− 0.38	− 1.43	0.67	0	0.48
Mean degree of ICA stenosis n %	40/223	4	5.06	0.86	9.27	62	0.02
BMI	59/170	4	− 0.33	− 1.34	0.68	0	0.52

Risk factors	No. of patients (yes/no)	No. of included studies	ORs	Lower 95% CIs	Upper 95% CIs	*I*^2^ (%)	*P* value
Sex (male)	2528/931	17	0.97	0.80	1.16	0	0.71
Diabetes	471/1266	17	1.26	0.97	1.64	0	0.08
Hypertension	1291/529	16	1.07	0.82	1.39	15	0.62
Previous MI	242/705	4	0.92	0.43	1.98	39	0.83
Dyslipidaemia	432/419	10	1.13	0.73	1.75	0	0.59
Statin use	334/731	7	0.54	0.41	0.71	0	< 0.0001
Smoking	676/367	7	1.06	0.78	1.44	0	0.69
Contralateral stenosis	136/261	6	1.06	0.71	1.59	0	0.76
Pre-operative symptoms	820/847	14	1.02	0.80	1.31	0	0.86
Hyperperfusion	64/431	6	38.67	19.32	77.38	0	< 0.0001
Selective shunting use	11/251	2	0.93	0.11	7.80	0	0.95

*CI* confidence interval; *MD* mean difference; *OR* odds ratio; *POCD* postoperative cognitive dysfunction

**Fig. 3:**
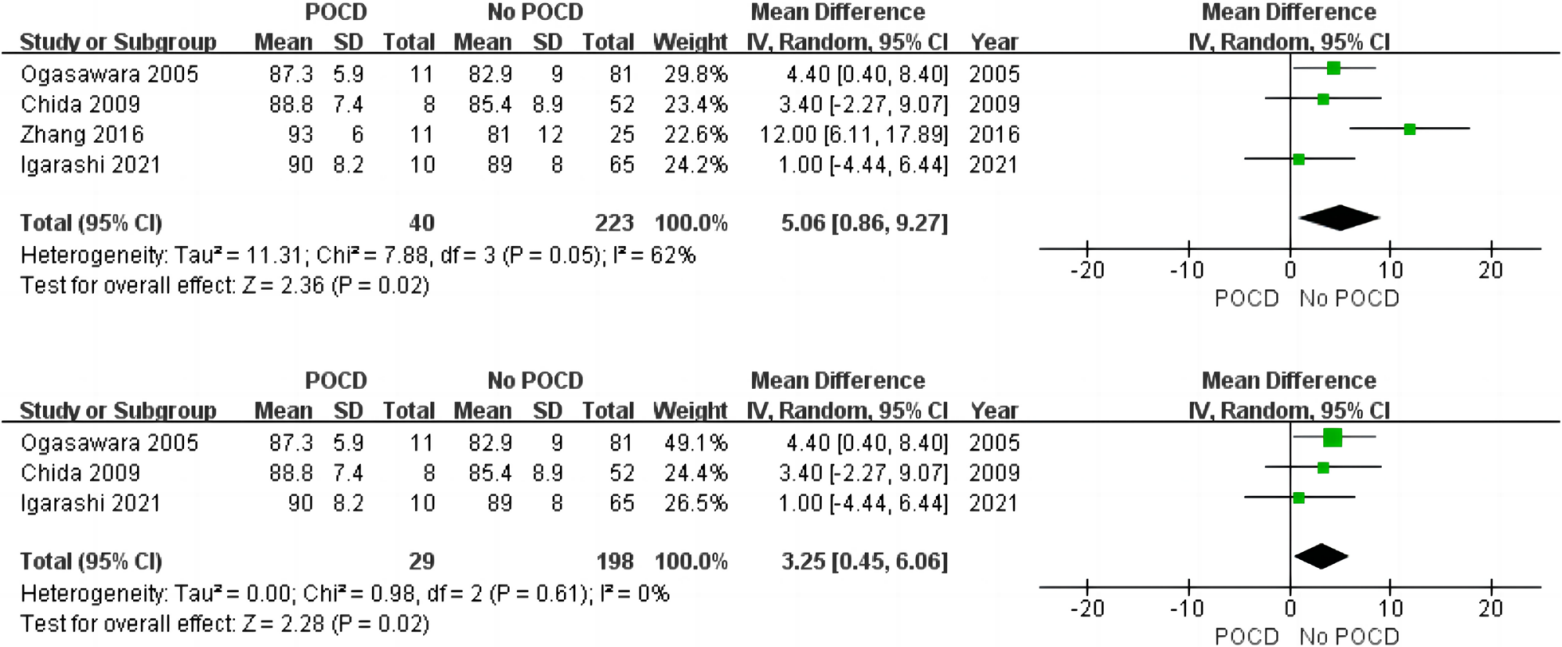
Forest plot and sensitivity analysis of studies (n = 4) reporting mean degree of ICA stenosis in patients undergoing CEA

**Fig. 4:**
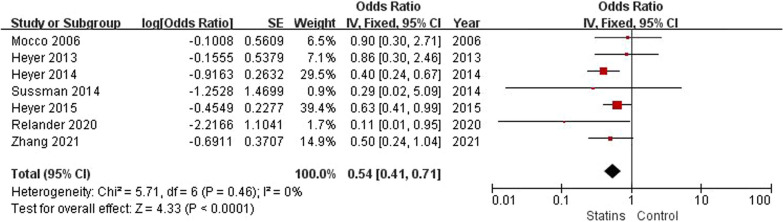
Forest plot of studies (n = 7) reporting preoperative use of statins in patients undergoing CEA

**Fig. 5:**
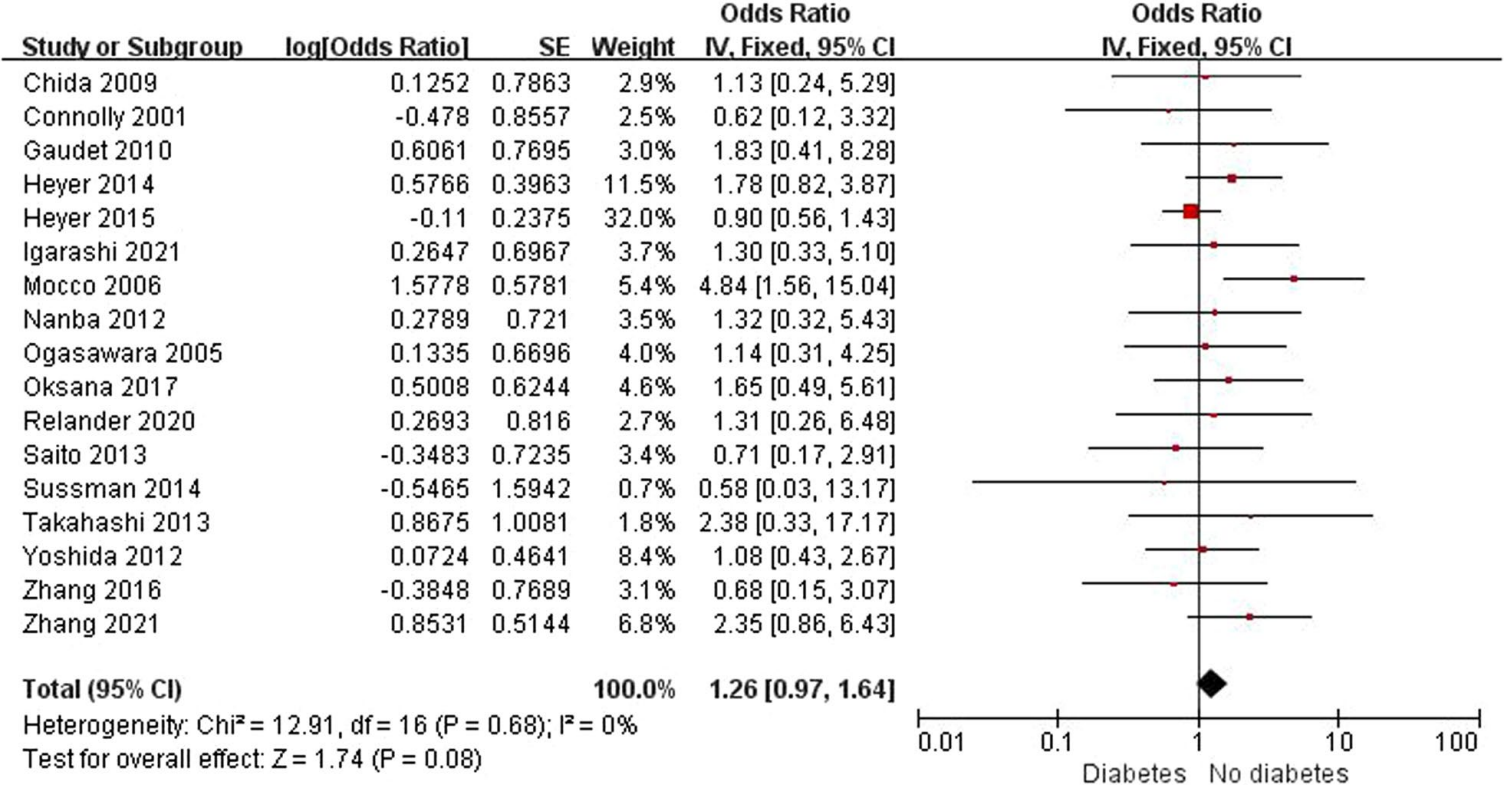
Forest plot of studies reporting diabetes (n = 17) in patients undergoing CEA

**Fig. 6:**
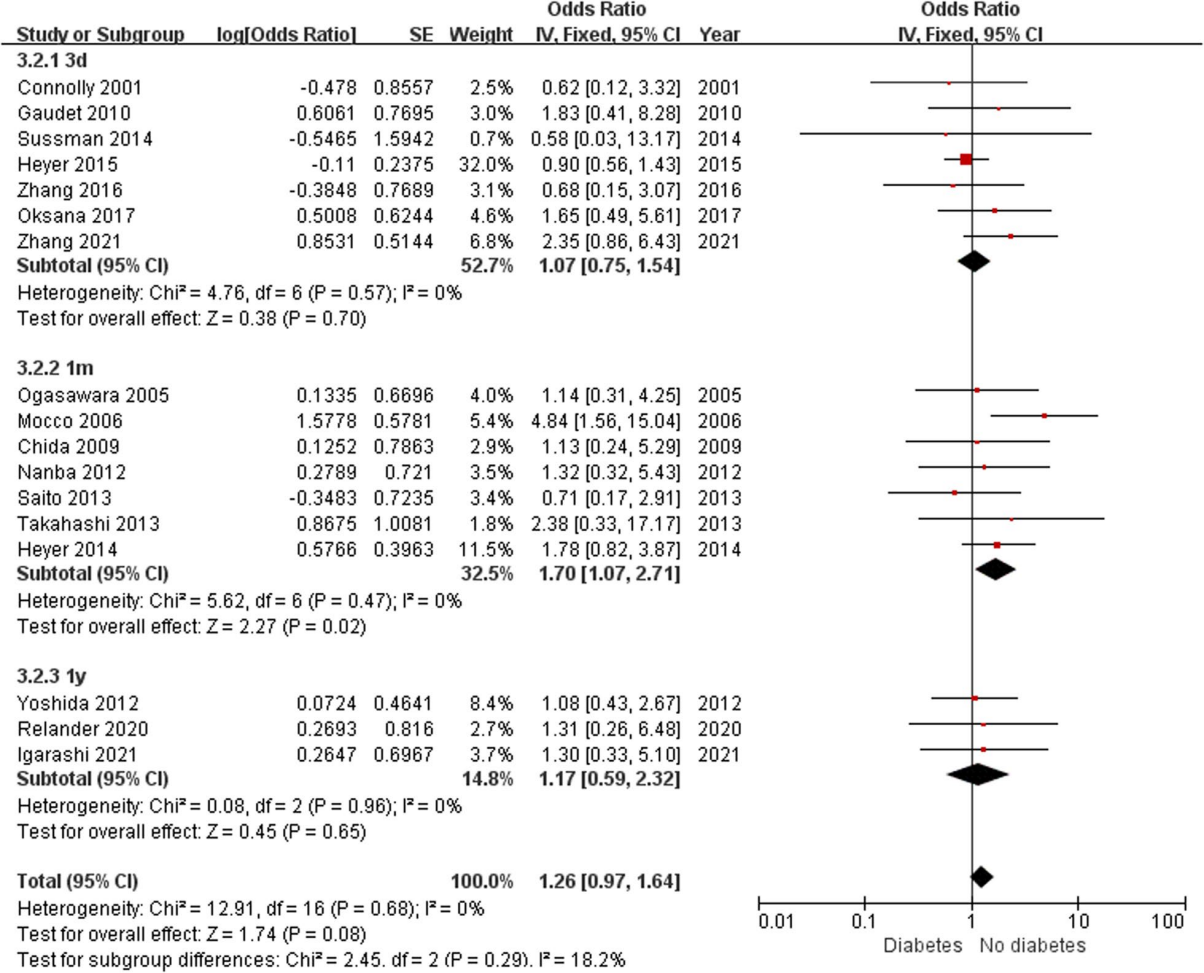
Forest plot of subgroup analysis of studies reporting diabetes (n = 17) in patients undergoing CEA

Patient-related risk factors were demonstrated in 18 observational articles (shown in Table 1 and Table 2). The results revealed that the degree of ICA stenosis (OR: 5.06, 95% CI 0.86–9.27) could be the potential risk factor for POCD. Although the result lacked credibility because its heterogeneity was significant (*I*^2^ = 62%, *P* = 0.02), subsequent sensitivity analysis revealed that the degree of ICA stenosis was still a risk factor for POCD (OR: 3.25, 95% CI 0.45–6.06, *I*^2^ = 0%, *P* = 0.02), as shown in Fig. 3. Besides, patients taking statins preoperative had a lower risk of POCD (OR: 0.54, 95% CI 0.41—0.71, *I*^2^ = 0%, *P* < 0.001), as illustrated in Fig. 4. No significant differences were founded in age, sex, BMI, education years, diabetes, dyslipidemia, previous MI, hypertension, smoking, contralateral stenosis and preoperative symptoms, ORs and 95%CI were summarized in Table 2. The subgroup analysis indicated that diabetes could significantly increase the incidence of POCD at 1 month after surgery. (OR: 1.70, 95% CI 1.07—2.71, *I*^2^ = 0%, *P* = 0.02), as shown in Figs. 5 and 6.

### Procedure-related risk factors for POCD

**Fig. 7:**
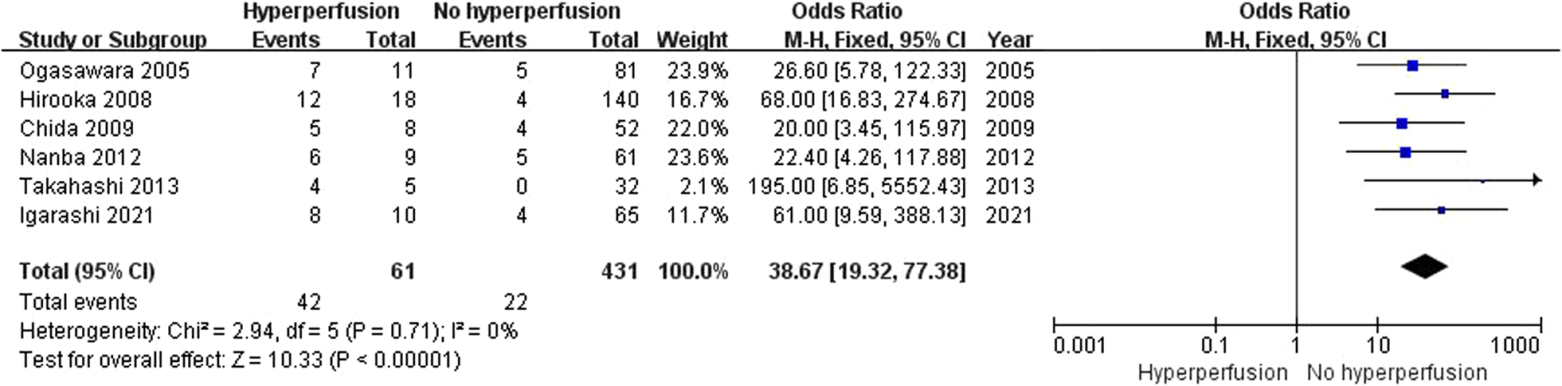
Forest plot of studies (n = 6) reporting presence/absence of hyperperfusion in patients undergoing CEA

There were 6 articles reporting hyperperfusion, 2 articles evaluating selective shunting placement and 9 studies assessing cross-clamping duration. The result of our analysis indicated that the risk of POCD increased in patients with hyperperfusion after CEA (OR: 38.67, 95% CI 19.32–77.38, *I*^2^ = 0%, *P* < 0.00001) as shown in Table 2 and Fig. 7. Selective shunting placement and cross-clamping duration were not significant risk factors for POCD in patients following CEA.

### Publication bias assessment

The Egger's coefficient bias of POCD incidence (*P* = 0.3) and each factor relevant studies did not indicate the presence of publication bias: age (*P* = 0.891), sex (*P* = 0.939), diabetes (*P* = 0.518), dyslipidemia (*P* = 0.699), hypertension (*P* = 0.913), preoperative symptoms (*P* = 0.092), and cross-clamping duration (*P* = 0.299) ( The result of Egger's test details can be found in Additional file 1^1^ Appendix 2).

## Discussion

Postoperative cognitive dysfunction is a severe neurological complication after carotid endarterectomy and contributes to a variety of adverse outcomes, such as longer hospital stays and decrease in life quality [14]. However, there is no evidence indicating that specific treatments could cure POCD currently. Early identification of relative factors might play a vital role in reducing the incidence of POCD after CEA. Compared to the meta-analysis of 2020 [9], we included 7 additional studies published since then and added data from an additional 739 patients. Besides, we added some indicators that might be related to POCD, such as education years, degree of ICA stenosis and others. Finally, a total number of 3459 patients with CEA and 16 risk factors based on 22 studies were identified for meta-analysis. The potential risk factors of POCD were hyperperfusion and degree of ICA stenosis, while diabetes could significantly increase the incidence of POCD 1 month after surgery. What’s more, preoperative statin use could be a protective factor for POCD following CEA.

In the present article, the pooled incidence of POCD after CEA was 19% (95% CI 0.16–0.24), which was consistent with the result in a previous study (6–36%) [4]. Similar to previous studies, the heterogeneity of the synthetic incidence was significant (*I*^2^ = 93.1%, *P* < 0.001), which might be related to the adjustment for confounding factors in the most of included studies [15, 16]. Furthermore, we conducted subgroup analysis, which revealed the incidence of POCD at 3 days, 1 month, 1 year after CEA were 32%, 13% and 13% respectively. However, another meta-analysis reported the incidence of cognitive decline at 1 month (20.5%) was higher than our result, which might be related to baseline characteristics, sample size and diagnostic criteria in the included studies [9].

The result of this article revealed that hyperperfusion was a significant risk factor for POCD after CEA. Cerebral hyperperfusion, a dramatic increase in cerebral blood flow (CBF) exceeded the metabolic requirements of the brain tissue, could cause cerebral oedema or intracerebral haemorrhage. Hyperperfusion post-CEA was defined as a 100% increase or greater in CBF compared with preoperative values. CBF was measured by single-photon emission computerized tomography (SPECT) scanning before and after CEA in included articles. Some studies indicated that asymptomatic hyperperfusion could also lead to postoperative cortical neural damage and resulted in cognitive decline [17, 18]. There are several possibilities to explain the relationship of hyperperfusion and cognitive decline after CEA. Firstly, T2-weighted MR imaging on CBF showed hyperintense lesions in the region corresponding to hyperperfusion, which indicated the existence of cytotoxic edema [19]. Secondly, significant cerebral ischemia caused by embolism or clamping of the ICA during CEA can contribute to reperfusion hyperemia [10].

Moreover, different from the meta-analysis of 2020 [9], we found that the degree of ICA stenosis could be another risk factor for POCD after CEA. Although the result lacked credibility because of its significant heterogeneity (*I*^2^ = 62%, *P* = 0.02), subsequent sensitivity analysis revealed that the degree of ICA stenosis was still a significant risk factor for POCD. We found the study by Zhang et al. [20] might have a major impact on the heterogeneity in the result, which might be associated with the inconsecutive subjects and the small sample in the study. The main mechanism by carotid stenosis contributed to POCD might be related to the impaired cognition before surgery caused by hypoperfusion [21]. Previous study indicated that patients with unilateral carotid stenosis of 70% produced poorer cognitive function such as verbal memory and executive function, which was likely attributed to chronic hypoperfusion and microemboli from unstable carotid plaques, while cognitive impairment preoperative had been proved to be related to POCD [22, 23]. Besides, cerebral hypoperfusion induced by carotid stenosis was also thought to accelerate amyloid and tau deposition, which might be another potential link with POCD [24].

From this meta-analysis, We did not find significant relationships between patient-related factors and POCD, such as age, sex, hypertension and others. Age is a well known risk factor for POCD in patients undergoing cardiac and noncardiac surgery [25, 26]. The discrepancy from the current findings might be due to the fact that participants included in identified studies were all elderly. Besides, as previously mentioned, cross-clamping duration was an independent risk factor for POCD [20]. Conversely, our meta‐analysis showed that cross-clamping duration was not related to the occurrence of POCD after CEA, which may be associated with the adjustment for confounders.

Although there were no significant differences in diabetes, the subgroup analysis indicated that diabetes could significantly increase the incidence of POCD 1 month after surgery. Similarly, in a retrospective review of more than 6000 CEA patients, Tu et al. [27] verified that diabetes independently predicts stroke or death within 30 days of surgery. However, the association between diabetes and POCD is controversial. Diabetes, analyzed by HbA1c, was considered as a predisposing factor for POCD in several studies [28, 29], while others did not [4, 8]. Although the exact mechanism was unclear, glycemic variables was considered to play an important role in developing POCD [30]. Hyperglycemia is both a cause and result of inflammation, while neuroinflammation is an important mechanism for development of POCD, which may explain the relationship between hyperglycemia and POCD [31, 32].

Another interesting result demonstrated that statin use preoperative was a protective factor for POCD following CEA, which consistent with conclusions of previous meta-analysis [9]. Statins, known as lipid-lowering drugs, might reduce POCD risk due to lower cholesterol levels. Nevertheless, there was no significant association between dyslipidaemia and POCD in our study. According to the pleiotropic effect of statins, we speculated that statins might exert neuroprotective effects through other pathways. A previous research suggested that statins play a neuroprotective role through anti-inflammatory and regulating nitric oxide production to attenuate the ischemic reperfusion injury of asymptomatic patients after CEA [33]. In the present article, we did not analyze the effects of different types and duration of statin use on POCD. Therefore, it would be interesting to explore the effect of pre-operative treatment duration with statins on prevention of POCD after CEA.

There are some limitations in the current meta-analysis. Firstly, the absence of standardisation to define POCD may limit the precision of the analysis. Secondly, the time of evaluating cognitive function is only within 1 year after CEA in included studies, which may lead to errors in the incidence of POCD. Finally, some other risk factors such as frailty, anesthesia method and data on middle cerebral artery Doppler are not considered in this study.

## Conclusions

The potential risk factors of POCD were hyperperfusion and the degree of ICA stenosis, while diabetes could significantly increase the incidence of POCD 1 month after surgery. What’s more, preoperative statin use could be a protective factor for POCD following CEA. In order to develop accurate prevention strategies, surgeons and nursing staff should have a comprehensive understanding of POCD after CEA. This meta-analysis can help them systematically identify risk factors.

### Supplementary Information

**Additional file 1**: Search Strategy and Supplemental Tables and Figures.

## Data Availability

The original contributions presented in the study are included in the article/Suplementary Material; further inquiries can be directed to the corresponding author.

## Abbreviations

POCD: Postoperative cognitive dysfunction
CEA: Carotid endarterectomy
ORs: Odds ratios
MDs: Mean differences
NOS: Newcastle–Ottawa Scale
CI: Confdence interval
ICA: Internal carotid artery
CAS: Carotid artery stenting
BMI: Body mass index
CBF: Cerebral blood flow
