# Supplementary material for: The risk factors of postoperative cognitive dysfunction in patients undergoing carotid endarterectomy: an updated meta-analysis
Source: J Cardiothorac Surg. 2023 Nov 9;18:309. doi: 10.1186/s13019-023-02428-6 (PMC10636909; doi:10.1186/s13019-023-02428-6)
Supplement: Supplementary file 1 — Additional file 1: Search Strategy and Supplemental Tables and Figures. [file 13019_2023_2428_MOESM1_ESM.doc]

# *Supplementary materials*

# The risk factors of postoperative cognitive dysfunction in patients undergoing carotid endarterectomy: an updated meta-analysis

Jinhua He^1^, Ran Duan^1^, Peng Qiu^2^, Huanhuan Zhang^1^, Meng Zhang^1^, Meinv Liu^1^, Xiaoqian Wu^1^, Jianli Li^1*^

*** Correspondence:** Jianli Li: hblijianli[@163.com](mailto:hblijianli123@163.com)

# Appendix 1: **Search Strategy**

**PubMed:**

(((Cognition[MeSH Terms]) OR (Cognitions[Title/Abstract] OR Cognitive Function[Title/Abstract] OR Cognitive Functions[Title/Abstract] OR Function, Cognitive[Title/Abstract] OR Functions, Cognitive[Title/Abstract])) OR ((Cognitive Dysfunction[MeSH Terms]) OR (Cognitive Dysfunctions[Title/Abstract] OR Dysfunction, Cognitive[Title/Abstract] OR Dysfunctions, Cognitive[Title/Abstract] OR Cognitive Impairments[Title/Abstract] OR Cognitive Impairment[Title/Abstract] OR Impairment, Cognitive[Title/Abstract] OR Impairments, Cognitive[Title/Abstract] OR Mild Cognitive Impairment[Title/Abstract] OR Cognitive Impairment, Mild[Title/Abstract] OR Cognitive Impairments, Mild[Title/Abstract] OR Impairment, Mild Cognitive[Title/Abstract] OR Impairments, Mild Cognitive[Title/Abstract] OR Mild Cognitive Impairments[Title/Abstract] OR Mild Neurocognitive Disorder[Title/Abstract] OR Disorder, Mild Neurocognitive[Title/Abstract] OR Disorders, Mild Neurocognitive[Title/Abstract] OR Mild Neurocognitive Disorders[Title/Abstract] OR Neurocognitive Disorder, Mild[Title/Abstract] OR Neurocognitive Disorders, Mild[Title/Abstract] OR Cognitive Decline[Title/Abstract] OR Cognitive Declines[Title/Abstract] OR Decline, Cognitive[Title/Abstract] OR Declines, Cognitive[Title/Abstract] OR Mental Deterioration[Title/Abstract] OR Deterioration, Mental[Title/Abstract] OR Deteriorations, Mental[Title/Abstract] OR Mental Deteriorations[Title/Abstract]))) AND ((Endarterectomy, Carotid[MeSH Terms]) OR (Carotid Endarterectomy[Title/Abstract] OR Carotid Endarterectomies[Title/Abstract] OR Endarterectomies, Carotid[Title/Abstract]))

**Cochrane:**

#1: MeSH descriptor: [Cognitive Dysfunction] explode all trees

#2: (Impairments, Mild Cognitive or Neurocognitive Disorders, Mild or Mild Neurocognitive Disorder or Mild Neurocognitive Disorders or Cognitive Impairment, Mild or Impairment, Mild Cognitive or Disorders, Mild Neurocognitive or Cognitive Impairments, Mild or Neurocognitive Disorder, Mild or Mild Cognitive Impairment or Disorder, Mild Neurocognitive or Mild Cognitive Impairments or Decline, Cognitive or Deteriorations, Mental or Declines, Cognitive or Cognitive Decline or Mental Deteriorations or Cognitive Declines or Mental Deterioration or Deterioration, Mental or Dysfunction, Cognitive or Cognitive Impairments or Impairments, Cognitive or Cognitive Dysfunctions or Impairment, Cognitive or Cognitive Impairment or Dysfunctions, Cognitive):ti,ab,kw

#3: MeSH descriptor: [Cognition] explode all trees

#4: (Cognitions or Functions, Cognitive or Function, Cognitive or Cognitive Functions or Cognitive Function):ti,ab,kw

#5: #1 or #2 or #3 or #4

#6: MeSH descriptor: [Endarterectomy, Carotid] explode all trees

#7: (Carotid Endarterectomy or Carotid Endarterectomies or Endarterectomies, Carotid):ti,ab,kw

#8: #6 or #7

#9：#5 and #8

**Embase:**

('carotid endarterectomy'/exp OR 'carotid endarterectomy' OR (('carotid'/exp OR carotid) AND ('endarterectomy'/exp OR endarterectomy))) AND ('cognitive decline'/exp OR 'cognitive decline' OR (cognitive AND ('decline'/exp OR decline)) OR 'cognition'/exp OR cognition OR 'cognitive defect'/exp OR 'cognitive defect' OR (cognitive AND defect) OR 'neurocognitive impairment'/exp OR 'neurocognitive impairment' OR (neurocognitive AND ('impairment'/exp OR impairment)) OR 'postoperative cognitive dysfunction'/exp OR 'postoperative cognitive dysfunction' OR (postoperative AND cognitive AND dysfunction))

# Appendix 2: Supplementary Figures

**
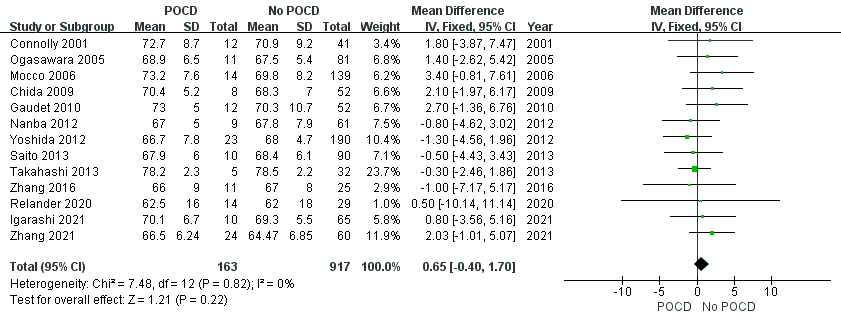
**

**Supplementary Figure 1.** Forest plot of studies reporting age (n=13) in years.


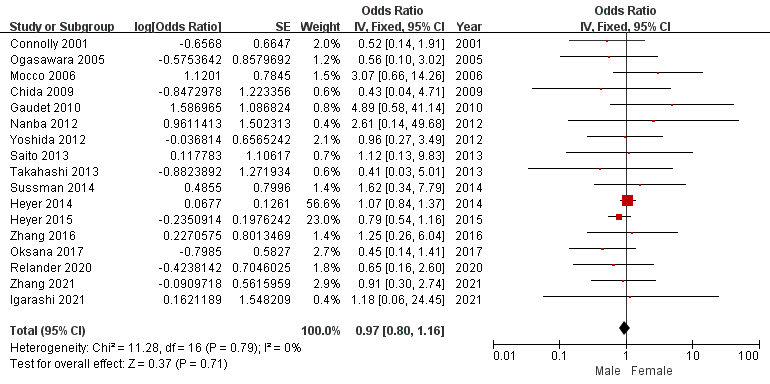


**Supplementary Figure 2.** Forest plot of studies reporting gender (n=17).


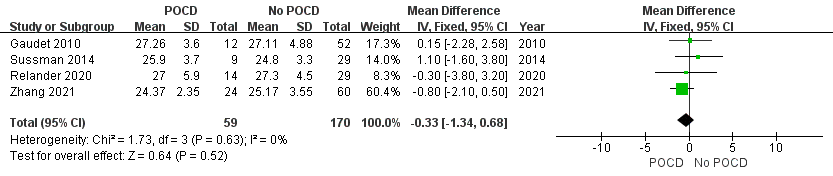


**Supplementary Figure 3.** Forest plot of studies reporting BMI (n=4).


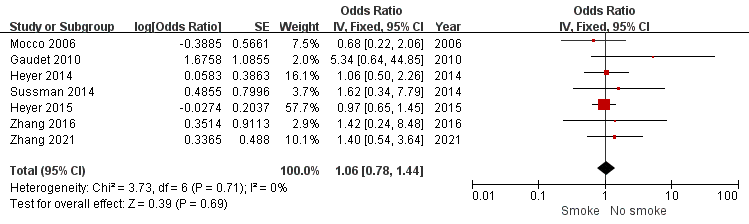


**Supplementary Figure 4.** Forest plot of studies reporting smoking (n=7)


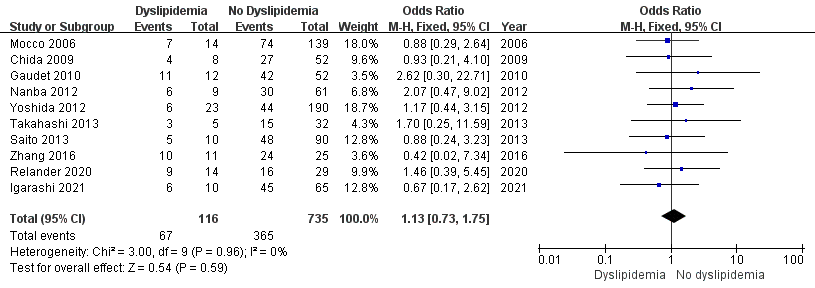


**Supplementary Figure 5.** Forest plot of studies reporting dyslipidemia (n=10)


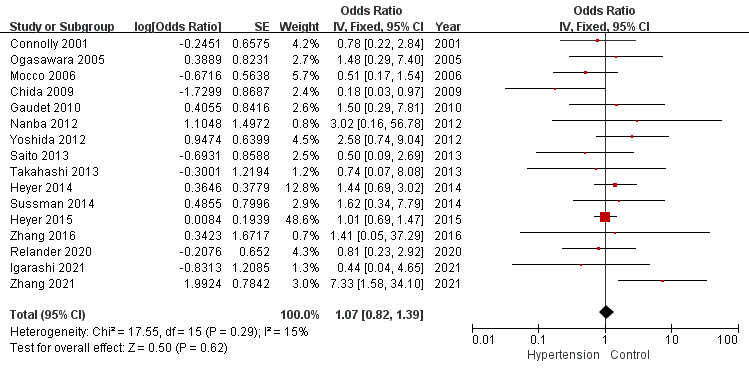


**Supplementary Figure 6.** Forest plot of studies reporting hypertension (n=16).


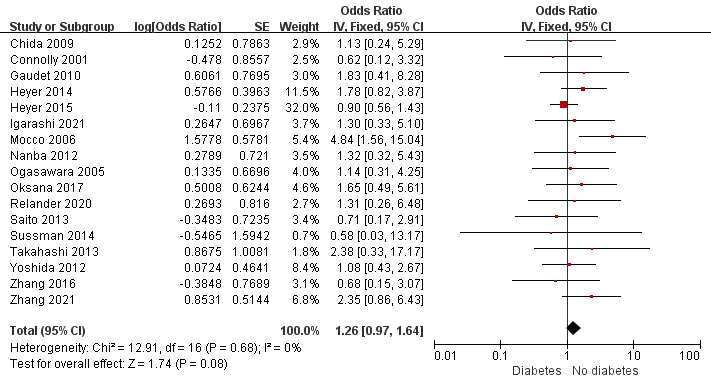


**Su****pplementary Figure 7.**  Forest plot of studies reporting diabetes (n=17).


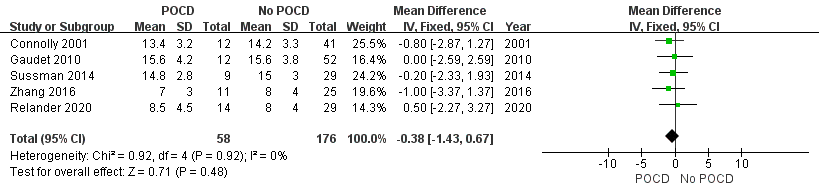


**Supplementary Figure 8.**  Forest plot of studies reporting education years (n=5).


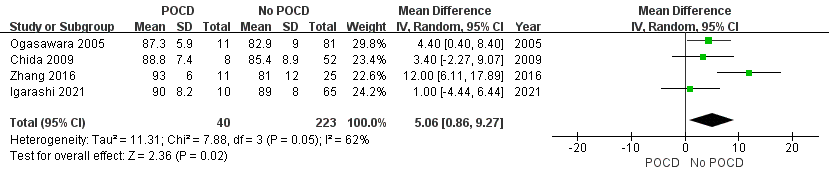


**Supplementary Figure 9.** Forest plot of studies reporting mean degree of ICA stenosis (n=4).


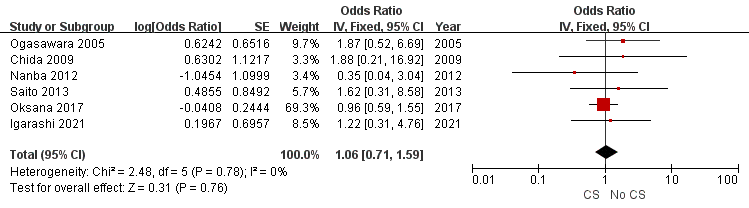


**Supplementary Figure 10.** Forest plot of studies reporting contralateral stenosis (n=6).


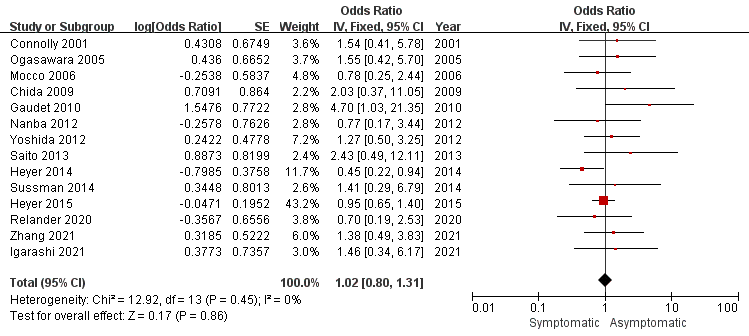


**Supplementary Figure 11.** Forest plot of studies reporting pre-operative symptoms (n=14).


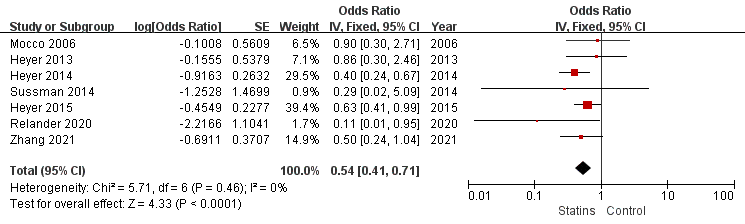


**Supplementary Figure 12.** Forest plot of studies reporting Statin use (n=7).


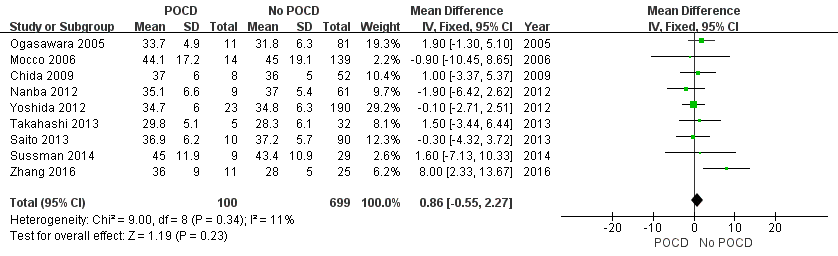


**Supplementary Figure 13.** Forest plot of studies reporting time of cross-clamping (n=9).


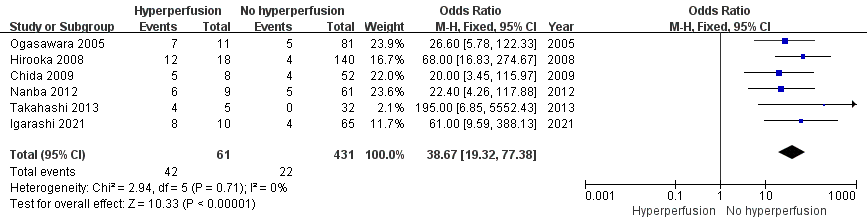


**Supplementary Figure 14.**  Forest plot of studies reporting hyperperfusion (n=6).


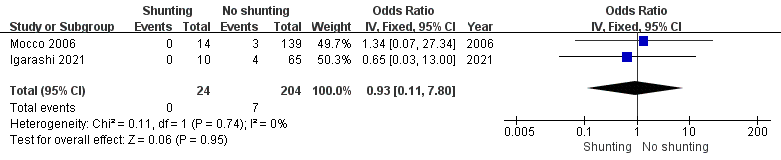


**Supplementary Figure 15.** Forest plot of studies reporting selective shunting use (n=2).


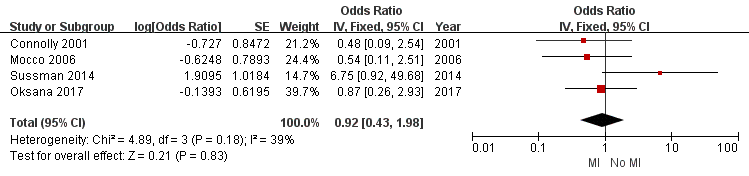


**Supplementary Figure 16.** Forest plot of studies reporting Previous MI (n=4).


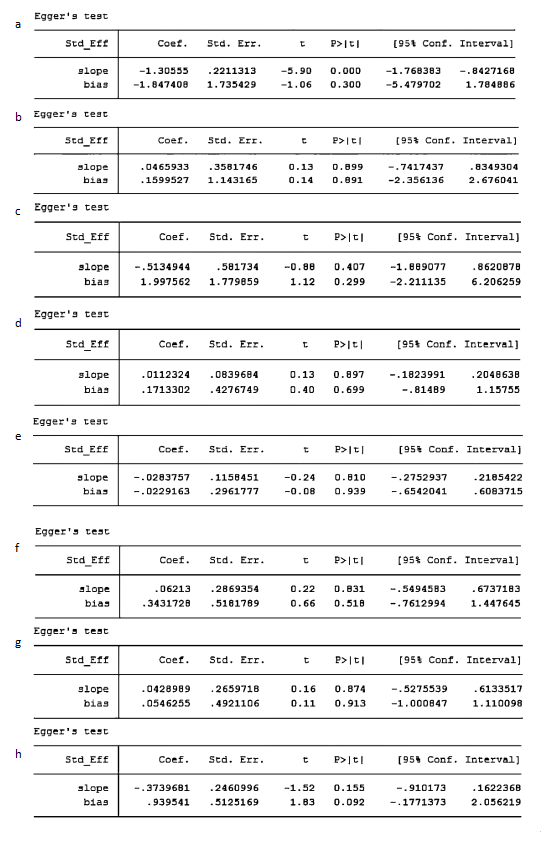


**Supplementary Figure 17.** The Egger's coefficient bias of POCD incidence and each factors relevant studies. (a. Incidence of POCD; b. Age; c. Cross-clamping; d. Dyslipidaemia; e. Sex; f. Diabetes; g. Hypertension; h. Pre-operative symptoms.)

## Appendix 3: Supplementary Tables

**Supplementary Table 1.** Neuropsychological assessment used in the observational studies on POCD after carotid endarterectomy.

| First author, year | Neuropsychological Assessment |
| --- | --- |
| Zhang, 2021 | - Mini Mental State Examination (MMSE) |
| Igarashi, 2021 | - Wechsler Adult Intelligence Scale-Revised (WAIS-R) - Wechsler Memory Scale (WMS) - Rey-Osterrieth Complex Figure Test (RCFT) |
| Relander, 2020 | - Rivermead Behavioral Memory test - Auditory verbal learning test - Rey visual learning test - Digit span forwards and backwards - Letter Cancellation test (time to complete) - Trail Making test, part B subtracted by part A - Stroop test |
| Robison, 2019 | - Controlled Oral Word Association Test (COWAT) - Hopkins Verbal Learning Test (HVLT) - Buschke Selective Reminding Test (SRT) - ReyOsterrieth Complex Figure Test (ROCF) - Trails Making Test (TMT) - Rapid Finger Tapping Test - Grooved Pegboard Test |
| Oksana, 2017 | - Mini Mental State Examination (MMSE) |
| Zhang, 2016 | - Clock Drawing Task (CDT) - Mini Mental State Examination (MMSE) |
| Heyer, 2015 | - Buschke Selective Reminding Test - Controlled Oral Word Association Test (COWAT) - Finger Tapping Test - Grooved Pegboard - Halstead–Reitan Trails making (A and B) - Hopkins Verbal Learning Test - Rey-Osterrieth Complex Figure (Copy and Recall) |
| Heyer, 2014 | - Buschke Selective Reminding Test (SRT) - Controlled Oral Word Association Test (COWAT) - Finger Tapping Test - Grooved Pegboard - Halstead–Reitan Trails making (A and B) - Hopkins Verbal Learning Test - Rey-Osterrieth Complex Figure (Copy and Recall) |
| Sussman, 2014 | - Boston Naming Test - Controlled Oral Word Association (COWA) - Halstead–Reitan Trails making (A and B) - Rey–Osterreith Complex Figure test - copy portion |
| Heyer, 2013 | - Hopkins Verbal Learning Test - Buschke Selective Reminding Test (SRT) - Controlled Oral Word Association Test (COWAT) - Rey-Osterrieth Complex Figure (Copy and Recall) - Grooved Pegboard - Finger Tapping Test - Halstead–Reitan Trails making (A and B) |
| Saito, 2013 | - Rey-Osterreith Complex Figures (Copy and Recall) - Wechsler Adult Intelligence Scale III (WAIS-III)-Verbal and Performance - Wechsler Memory Scale (WMS) -memory and attention |
| Takahashi, 2013 | - Rey-Osterreith Complex Figures (Copy and Recall) - Wechsler Adult Intelligence Scale III (WAIS-III)-Verbal and Performance - Wechsler Memory Scale (WMS)-memory and attention |
| Yoshida, 2012 | - Wechsler Adult Intelligence Scale- verbal and performance IQ - Wechsler Memory Scale (WMS)- memory and attention - Rey-Osterreith Complex Figures |
| Nanba, 2012 | - Wechsler Adult Intelligence Scale Revised (WAIS-R) - Wechsler Memory Scale (WMS) - Rey-Osterreith Complex Figure test |
| Gaudet, 2010 | - Boston Naming Test - Controlled Oral Word Association (COWA) - Grooved Pegboard - Halstead–Reitan Trails making (A and B) - Hopkins Verbal Learning - Rey-Osterrieth Complex Figure test |
| Chida, 2009 | - Rey-Osterreith Complex Figure test - Wechsler Adult Intelligence Scale Revised (WAIS-R) - Wechsler Memory Scale (WMS) |
| Soinne, 2009 | - Boston Naming Test (BNT) - Corsi blocks - Visual span forwards and backwards (CB-VISP) - Halstead–Reitan Trails making (A and B) - Letter and category naming with 1-min generation of words by a letter (WF-L) or by a category (WF-C) - Letter Cancellation Task (LCT) - Purdue pegboard - Rey’s Auditory Verbal Learning Test (RAVLT) - Stroop Color-Word Task - Wechsler Adult Intelligence Scale Revised (WAIS-R) - Digit span forwards and back-wards |
| Hirooka, 2008 | - Rey–Osterreith Complex Figure test - Wechsler Adult Intelligence Scale Revised (WAIS-R) - Wechsler Memory Scale (WMS) |
| Mocco, 2006 | - Boston Naming Test - Controlled Oral Word Association (COWA) - Halstead–Reitan Trails making (A and B) - Rey–Osterreith Complex Figure – copy portion |
| Ogasawara, 2005 | - Wechsler Adult Intelligence Scale Revised (WAIS-R) - Wechsler Memory Scale (WMS) - Rey–Osterreith Complex Figure test |
| Sahlein, 2003 | - Halstead–Reitan Trails making (A and B) - Controlled Oral Word Association test (COWA) - Rey-Osterrieth Complex Figure - copy portion |
| Connolly, 2001 | - Halstead-Reitan Trails making (A and B) - Controlled Oral Word Association test(COWA) - Rey -Osterrieth Complex Figure - copy portion |

**Supplementary Table 2.** Risk of bias table for assessing the quality of cohort studies by using the Newcastle-Ottawa Scale.

|  | Representativenes of the exposed cohort | Selection of the non exposed cohort | Ascertainment of exposure | Demonstration that outcome of interest was not present at start of study | Comparability of cohorts on the basis of the gender | Comparability of cohorts on the basis of the age | Assessment of outcome | Was follow-up long enough for outcomes to occur | Adequacy of follow up of cohorts |
| --- | --- | --- | --- | --- | --- | --- | --- | --- | --- |
| Zhang, 2021 | * |  | * | * |  | * | * |  | * |
| Igarashi, 2021 | * |  | * | * |  |  | * | * | * |
| Relander, 2020 | * |  | * | * |  | * | * | * | * |
| Robison, 2019 | * | * | * | * |  | * | * |  | * |
| Oksana, 2017 | * |  | * | * |  | * | * |  | * |
| Zhang, 2016 |  | * | * | * | * | * | * | * | * |
| Heyer, 2015 | * | * | * | * | * | * | * |  | * |
| Heyer, 2014 | * | * | * | * | * | * | * | * |  |
| Sussman, 2014 | * | * | * | * |  |  | * |  | * |
| Heyer, 2013 | * |  | * | * |  | * | * | * | * |
| Saito,2013 | * |  | * | * |  |  | * | * | * |
| Takahashi, 2013 | * |  | * | * |  |  | * | * | * |
| Yoshida, 2012 | * |  | * | * |  |  | * | * | * |
| Nanba, 2012 | * |  | * | * |  |  | * | * | * |
| Gaudet, 2010 | * |  | * | * |  | * | * |  | * |
| Chida, 2009 | * |  | * | * |  |  | * | * | * |
| Soinne, 2009 | * |  | * | * | * | * | * | * | * |
| Mocco, 2006 | * |  | * | * |  | * | * | * |  |
| Ogasawara, 2005 | * |  | * | * |  |  | * | * | * |
| Sahlein, 2003 | * |  | * | * |  | * | * |  | * |
| Connolly, 2001 | * |  | * | * |  | * | * |  | * |
